# Clinical analysis and quality of life survey of hemophilia B patients in the central and western regions of China

**DOI:** 10.3389/fped.2024.1366990

**Published:** 2024-05-09

**Authors:** Wen Wang, Li Xu, Jingsheng Wu, Weiyong Liu, Jiao Jin, Jing Huang, Zhongjin Xu, Yali Huang, Bai Li, Yufeng Liu, Qing Zhang, Min Zhou, Jie Peng, Qun Hu

**Affiliations:** ^1^ Department of Pediatrics, Huazhong University of Science and Technology Tongji Medical College Tongji Hospital, Wuhan, China; ^2^ Department of Hematology, The Second People’s Hospital of Anhui Province, Hefei, China; ^3^ Department of Hematology, The First Affiliated Hospital of University of Science and Technology of China, Hefei, China; ^4^ Department of Ultrasound, The First Affiliated Hospital of University of Science and Technology of China, Hefei, China; ^5^ Department of Pediatrics, The Affiliated Hospital of Guizhou Medical University, Guiyang, China; ^6^ Department of Hematology, Jiangxi Provincial Children’s Hospital, Nanchang, China; ^7^ Department of Pediatrics, The First Affiliated Hospital of Zhengzhou University, Zhengzhou, China; ^8^ Haemophilia Diagnosis and Treatment Center, Department of Hematology and Oncology, Chengdu Women’s and Children’s Central Hospital, School of Medicine, University of Electronic Science and Technology of China, Chengdu, China; ^9^ Department of Hematology, Xiangya Hospital Central South University, Changsha, China

**Keywords:** hemophilia B, gene mutation, joint status, quality of life, carriers

## Abstract

**Objective:**

To study the current status of hemophilia B (HB) patients in the central and western regions of China.

**Methods:**

This cross-sectional, multicenter study was conducted in seven provinces in the central and western regions of China from April 2019 to June 2023. Samples were collected for the factor IX activity, inhibitor screen, and gene mutation. Furthermore, the status of six index joints and quality of life (QoL) were assessed.

**Results:**

A total of 185 HB patients (mild 15, moderate 75, and severe 95) with a median age of 12.17 years were enrolled. 30.3% (56/185) of patients had a family history of HB. 34.6% (64/185) of HB patients had diagnostic delay and 38.5% (69/179) experienced treatment delay. The incidence of inhibitors was 6.1% (11/179). We identified 123 genetic variants in this study, with missense mutations being the most common. 84.0% (89/106) of HB mothers were genetically identified as carriers, with 27.7% (13/47) of carriers having clotting factor levels less than 0.40 IU/ml. 71.4% (132/185) of HB patients had a history of joint hemorrhage, with a rate of target joint in these patients was 64.4% (85/132). Lower extremity joints were most often affected in patients. The Hemophilia Joint Health Score (HJHS) score was significantly positively correlated with the Hemophilia Early Arthropathy Detection with Ultrasound in China (HEAD-US-C) (*r* = 0.542, *P* < 0.001). Patients who received prevention treatment, inhibitor negative, without treatment delay, and without high-intensity replacement therapy showed a higher total score of the short form-36 health survey (SF-36).

**Conclusions:**

One-third of HB patients had delay in diagnosis and treatment, and the incidence of inhibitors was 6.1%. Target joints were present in nearly half of HB patients. Missense was the main mutation type. 84.0% of mothers of HB patients in this study were found to be carriers. HEAD-US-C and HJHS can complement each other in the evaluation of joint status and give a valid basis for early clinical management. Early detection and preventive treatment, as well as reducing high-intensity replacement therapy and inhibitor generation, can effectively improve the QoL of patients.

## 1 Introduction

Hemophilia B (HB) is an X-linked recessive inherited bleeding disorder, mostly affecting males, caused by deficiency of coagulation factor Ⅸ (FⅨ) due to mutations in the FⅨ gene (*F9*) (1). Defects in *F9* gene can also occur through spontaneous *de novo* mutations. While females are usually heterozygous for the gene mutation, known as hemophilia carriers (HCs). Lyonization is a common cause of decreased factor activity in HCs, leading to the occurrence of bleeding events (2). To date, the EAHAD database (https://f9-db.eahad.org) has listed 1,244 unique mutations in 4,713 HB individuals, of which point mutations are the most common (71.3%, 887/1,244), followed by deletion mutations (16.6%, 206/1,244) (3). Studies have shown that large deletion mutations, nonsense mutations and frameshift mutations are usually associated with severe phenotypes, while missense mutations can be associated with severe, moderate or mild HB (4).

The prevalence of HB in China is 0.5/100,000 (5). Based on “the seventh national population census” data, the population of the central and western regions of China is 74,754,657, so there are at least 3,738 HB patients exist in these areas (6). Bleeding in patients is usually related to FⅨ activity (FⅨ: C). Patients with mild HB (5%–40% of normal activity) may suffer delayed bleeding following trauma, surgery, or other invasive injuries, while patients with severe HB (<1% of normal activity) are at highest risk of frequent spontaneous bleeding into the joints and muscles, which leads to synovitis and arthropathy. Moderate HB (1%–5% of normal activity) is associated with less bleeding after relatively mild injury, although some patients can also have a clinically severe bleeding phenotype and musculoskeletal complications (7, 8). Joint bleeding accounts for 70%–80% of all bleeding and is more common in large synovial joints such as the elbow, knee, ankle, hip, and shoulder (9). Repeated joint bleeding can lead to the formation of target joint, resulting in decreased mobility of HB patients, corresponding muscle atrophy and joint dysfunction, and eventually lead to disability (9–11). Joint status can be evaluated clinically and by ultrasound. The Hemophilia Early Arthropathy Detection with Ultrasound in China (HEAD-US-C) is based on HEAD-US and adds two activity indicators of joint effusion and synovial vascular hyperplasia, which is highly sensitive to subclinical bleeding (12, 13).

Replacement therapy is currently the primary treatment for hemophilia in China, with therapeutic drugs including plasma, prothrombin complex concentrates (PCC), standard half-life concentrates (plasma derived FⅨ (pdFⅨ) and recombinant FⅨ (rFⅨ)), and extended half-life (EHL) concentrates. The Chinese guidelines on the treatment of hemophilia (version 2020) (14) state that rFⅨ or virus-inactivated PCC is the first treatment choice for HB, and fresh frozen plasma (FFP) can be used when these are not available. Regular prophylaxis to keep FⅨ: C levels above 1% is the gold standard of care for people with severe HB in China. Some patients did not, however, properly adhere to regular treatment due to financial and compliance issues (15). Additionally, during the period of replacement therapy, some individuals may develop neutralizing antibodies (inhibitor) to clotting factor concentrates (CFCs), making treatment ineffective and increasing the costs and psychological burdens (16). Delays in treatment can lead to severe joint damage and permanent disability, or even death if the bleeding involves major organs and/or the brain. The quality of life (QoL) of patients with hemophilia (PWH) is significantly affected due to the impact of frequent bleeding events, the financial and mental health burden. Ensuring the highest possible QoL for patients and their families is a major goal in the care of all chronic diseases (17, 18).

The primary objective of this study is to explore the clinical manifestations, genotype, joint status and QoL of HB patients in the real world in China, and to determine the carrier status of mothers of these HB patients and their factor Ⅸ level.

## 2 Materials and methods

### 2.1 Subjects and methods

From April 2019 to June 2023, this multicenter, cross-sectional study enrolled 185 HB patients (mild 15, moderate 75, and severe 95) from seven provinces in the central and western regions of China. Demographic data (age, sex, severity of HB, and family history) and clinical characteristics (age at diagnosis, age at first bleeding, diagnostic delay, causes of first bleeding, site of first bleed, annual bleeding rate (ABR), target joint, age at first treatment, first treatment modality, treatment delay, type of treatment (on-demand or prophylaxis), and high-intensity replacement therapy) were collected by review of medical records, organizing patient education activities, and conducting telephone follow-ups. Clinical assessments and ultrasound examinations were performed on the elbow, knee, and ankle joints. Questionnaires were administered to investigate the QoL of patients and, where relevant, their parents. Blood samples were taken from the HB patient to test factor activity, inhibitor, and gene mutation. Blood samples were taken from the mothers of HB patients for FIX level and mutation analysis. Informed consent was obtained from all study participants, and the study was approved by the Ethics Committees of Tongji Medical College of Huazhong University of Science and Technology.

### 2.2 Concept

2.2.1 Target joint: a single joint that had experienced bleeding more than three times within a consecutive 6-month period (15).

2.2.2 Replacement therapy: Treatment with exogenous coagulation factor to compensate for a deficiency of FⅨ in the body. Treatment after bleeding refers to “on-demand” treatment, while regular replacement therapy is called “prophylaxis” treatment (15).

2.2.3 High-intensity replacement therapy: replacement therapy for 3 consecutive days or more.

2.2.4 Inhibitor: Antibodies that developed to inhibit FⅨ. Inhibitor titer level ≥0.6 BU/ml is defined as inhibitor positive, which is classified as high-titer (titer level >5 BU/ml) and low-titer (titer level ≤5 BU/ml) (1).

2.2.5 Iatrogenic bleeding: Abnormal bleeding caused in the process of clinical diagnosis and treatment. For example, bleeding caused by surgery, injections, or blood drawing.

2.2.6 Diagnostic delay: age at first diagnosis minus age at first bleeding (19).

2.2.7 Treatment delay: age at first treatment minus age at first bleeding (19).

### 2.3 Instruments

2.3.1 Hemophilia Joint Health Score version 2.1 (HJHS 2.1) (20, 21).

The Chinese version of the HJHS 2.1 scale was used to evaluate the total joint health status of HB patients and overall gait, which is suitable for HB patients aged 4–18 years old. Each joint assessment included eight items and the highest score for each item ranges from 1 to 4 points, and the overall gait is worth 0–4 points. The highest score is 124 points, and the completely normal score is 0.

2.3.2 HEAD-US-C (22).

Ultrasound scores were referenced to the HEAD-US-C. The assessment of each joint included joint effusion, synovial hyperplasia, synovial vascular hyperplasia, cartilage, and bone (subchondral bone irregularities/osteophytes), with the highest score ranging from 2 to 4 points. The highest score of HEAD-US-C is 78, and the lowest score is 0.

2.3.3 The short form-36 health survey (SF-36) (age >14 years) (23).

SF-36v2 includes 8 dimensions: physical functioning (PF), role-physical functioning (RP), bodily pain (BP), general health (GH), vitality (VT), social functioning (SF), role-emotional functioning (RE), mental health (MH), and 1 reported health transition (HT). The sum of the eight dimensions is the comprehensive score, and the higher the score, the better the QoL.

2.3.4 Canadian hemophilia outcomes-kids’ life assessment instrument version2.0 (CHO-KLAT 2.0) (24).

The questionnaire includes the children's and parents' questionnaires, with a total of 35 questions. The questionnaire score is calculated by a specific tool, and the total score is 100, with higher scores indicating better QoL. Due to the cognitive differences of children, the children's questionnaire in this study is suitable for patients aged 8–14 years, and those younger than 8 years old are evaluated by parents.

### 2.4 Statistical analysis

SPSS 22.0 was used to analyze the data, and the count data were expressed as frequency (*n*) and percentage (%). Measurement data are expressed as median or mean ± standard deviation. Comparisons between two groups were made using the *t*-test, Pearson chi-square test, Fisher exact test or Mann-Whitney *U* test. The Pearson correlation analysis method was used to study the correlation, *P* < 0.05 was considered statistically significant.

## 3 Results

### 3.1 Demographic data

This study included a total of 185 HB patients (mild 15, moderate 75, and severe 95) and 106 HB mothers. Patients are all male, with a median age of 12.17 years. 30.3% (56/185) had a family history of HB. The demographic data are shown in Table 1.

**Table 1:** The demographic characteristics of 185 HB patients.

No. of patients		Mild (*N* = 15)	Moderate (*N* = 75)	Severe (*N* = 95)	Total (*N* = 185)
Gender	All male				
Age (years)	≤1	0 (0.0%)	2 (2.7%)	9 (9.5%)	11 (5.9%)
	1–3	1 (6.7%)	6 (8.0%)	14 (14.7%)	21 (11.4%)
	3–8	2 (13.3%)	18 (24.0%)	17 (17.9%)	37 (20.0%)
	8–14	3 (20.0%)	11 (14.7%)	21 (22.1%)	35 (18.9%)
	>14	9 (60.0%)	38 (50.7%)	34 (35.8%)	81 (43.8%)
Family history	Yes	5 (33.3%)	27 (36.0%)	24 (25.3%)	56 (30.3%)
	No	10 (66.7%)	48 (64.0%)	71 (74.7%)	129 (69.7%)

### 3.2 Clinical characteristics

The median age of first bleed was 1.00 years, 3.00 years for mild patients, 1.50 years for moderate, and 0.83 years for severe. 53.0% (98/185) of the first bleeding site was mucocutaneous hemorrhage. It was followed by joint hemorrhage and muscle hemorrhage, accounting for 16.2% (30/185) and 13.5% (25/185), respectively. Intracranial bleeding occurred in 5 patients (2.7%, 5/185). 111 instances of non-spontaneous bleeding were recorded, of which 87 (78.4%, 87/111) were traumatic bleeding and 24 (21.6%, 24/111) were iatrogenic bleeding. In traumatic bleeding, mucocutaneous hemorrhage accounted for the highest proportion (60.9%, 53/87), followed by muscle hemorrhage (21.8%, 19/87), joint hemorrhage (13.8%, 12/87), and intracranial hemorrhage (3.4%, 3/87). Spontaneous bleeding accounted for 40.0% (74/185) of first bleeds, including mucocutaneous hemorrhage (60.8%, 45/74), joint hemorrhage (24.3%, 18/74), muscle hemorrhage (8.1%, 6/74), intracranial hemorrhage (2.7%, 2/74), gastrointestinal hemorrhage (2.7%, 2/74), and urinary tract hemorrhage (1.4%, 1/74). The incidence of spontaneous bleeding was significantly different between non-severe patients and severe patients (*P* = 0.025).

The median age of diagnosis was 1.25 years old. 64 patients had diagnostic delay, with the median time of delay being 2.04 years. There was no noticeable variation in the time of diagnostic delay among HB patients with different severity (*P* = 0.217). The remaining 121 HB patients were diagnosed at (110/121) or before the first bleeding (11/121). 4 patients with a family history of HB were diagnosed at birth.

As shown in Table 2, 3.2% (6/185) of patients received no treatment. The median age of first treatment for the remaining 179 patients was 1.50 years. 38.5% (69/179) of HB patients experienced treatment delay, with a median delay time of 1.08 years. PCC was the most popular first-treatment drug (50.8%, 91/179), followed by plasma (39.1%, 70/179). Three patients received whole blood as their first-treatment drug, whereas four patients received cryoprecipitate. 57.5% (103/179) of HB patients received on-demand treatment and the remaining 76 HB patients received regular replacement therapy. The dose of CFCs (PCC, or FⅨ) for prophylaxis was 10–40 IU/kg once a week or 10–30 IU/kg twice a week. The prophylactic dose of plasma was 15–35 ml/kg once a week or 10–20 ml/kg twice a week, and the dose of PCC was 20–40 IU/kg once a week or 10–30 IU/kg twice a week. 50.8% (91/179) of patients had high-intensity replacement therapy for treatment of a bleeding. 6.1% (11/179) of HB patients were inhibitor-positive, with 6 severe (6.3%, 6/95) and 5 moderate patients (6.7%, 5/75), including 2 cases with high titer inhibitor. Among the 132 (71.4%, 132/185) patients who had previously had joint bleeding 64.4% (85/132) had target joints, of which 6 (7.1%, 6/85) HB patients were mild, 34 (40.0%, 34/85) were moderate, and 45 (52.9%, 45/85) were severe.

**Table 2:** The clinical characteristics of HB patients with different severity.

Clinical characteristics		Mild	Moderate	Severe	Total
Diagnostic delay	No. of patients	4	28	32	64
	Delayed time (year)	1.63	5.29	0.79	2.04
Age group					
≤14	No. of patients	2	8	17	27
	Delayed time (year)	0.67	0.96	0.42	0.50
>14	No. of patients	2	20	15	37
	Delayed time (year)	4.75	8.75	10.00	8.50
Treatment delay	No. of patients	3	33	33	69
	Time (year)	0.25	3.17	0.50	1.08
Age group					
≤14	No. of patients	2	11	17	30
	Delayed time (year)	0.21	1.00	0.33	0.46
>14	No. of patients	1	22	16	39
	Delayed time (year)	7.50	6.25	7.00	7.00
First treatment drug	PCC	8	44	39	91
	Plasma	6	24	40	70
	rFⅨ	0	4	7	11
	Cryoprecipitate	0	1	3	4
	Whole blood	0	2	1	3
	None	1	0	5	6
ABR	>6	8	42	59	109
	≤6	7	33	36	76
Joint bleeding	Yes	8	56	68	132
	No	7	19	27	53
Target joints	Yes	6	34	45	85
	No	9	41	50	100

PCC, prothrombin complex concentrate; rFIX, recombinant clotting factor IX; ABR, annual bleeding rate.

### 3.3 Assessment of joint status

#### 3.3.1 HJHS

**Figure 1:**
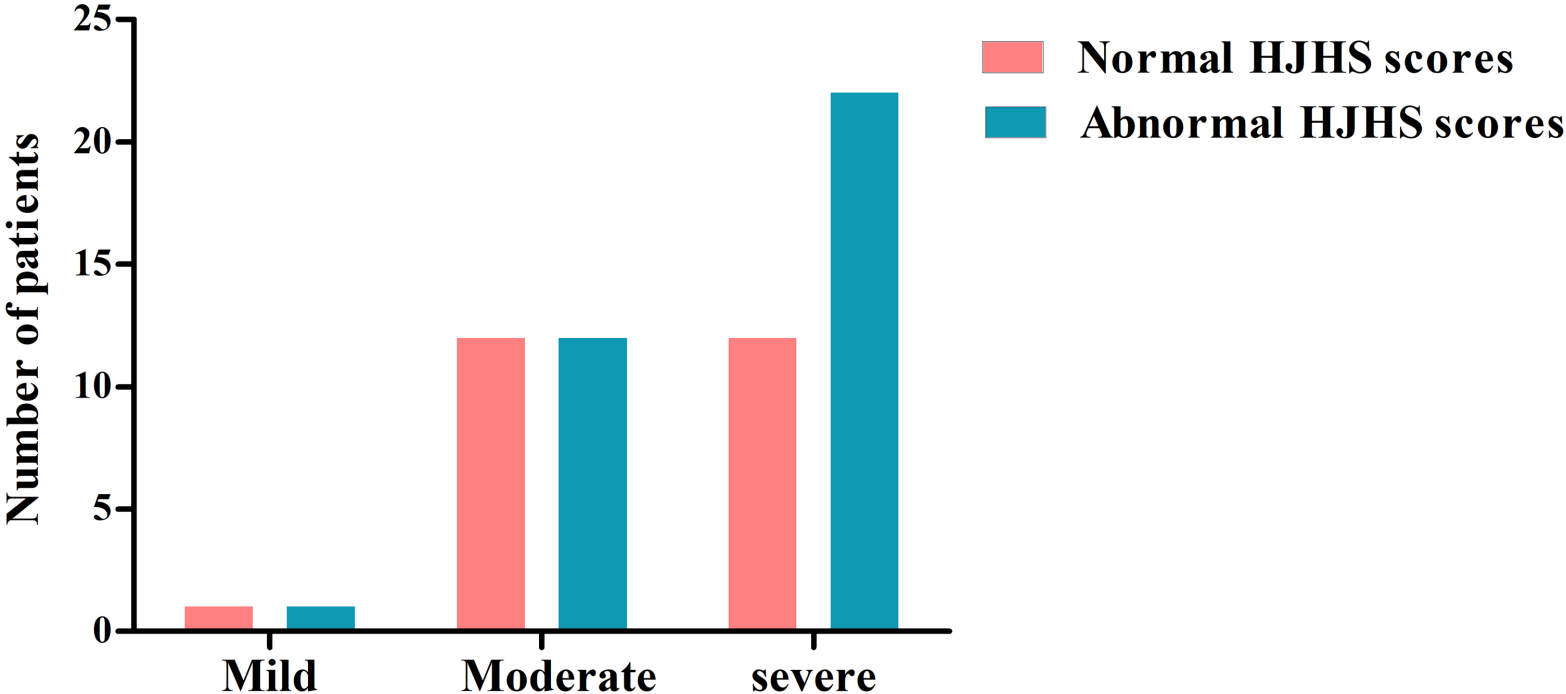
The distribution of patients with normal or abnormal HJHS scores in HB patients with different severity.

There were 78 HB patients aged 4–18 years in this study. Sixty HB patients, with a median age of 9.29 years, received HJHS examination. The number of patients with mild/moderate/severe HB with normal or abnormal HJHS scores is shown in the Figure 1. Twenty-five individuals with normal HJHS scores had a median age of 7.75 years, compared to 11.92 years old in 35 patients with an abnormal HJHS score. The difference was statistically significant (*P* = 0.002). The overall abnormal HJHS score ranged from 1 to 30 points, with an average of 11.91 points, and the mean (range) abnormal total joint score was 10.85 (1–28). Lower extremity joints (knee and ankle joints) were the most affected (Table 3).

**Table 3:** The distribution of pathological signs of each joint in 60 HB patients.

	Left elbow	Right elbow	Left knee	Right knee	Left ankle	Right ankle
Total	12 (20.0%)	13 (21.7%)	16 (26.7%)	18 (30.0%)	17 (28.3%)	19 (31.7%)
Swelling	6 (10.0%)	4 (6.7%)	10 (16.7%)	12 (20.0%)	6 (10.0%)	10 (16.7%)
Duration of swelling	3 (5.0%)	2 (3.3%)	5 (8.3%)	8 (13.3%)	3 (5.0%)	6 (10.0%)
Muscle atrophy	2 (3.3%)	3 (5.0%)	3 (5.0%)	6 (10.0%)	2 (3.3%)	1 (1.7%)
Crepitus on motion	2 (3.3%)	5 (8.3%)	11 (18.3%)	12 (20.0%)	9 (15.0%)	11 (18.3%)
Flexion loss	5 (8.3%)	3 (5.0%)	5 (8.3%)	8 (13.3%)	10 (16.7%)	7 (11.7%)
Extension loss	4 (6.7%)	3 (5.0%)	4 (6.7%)	6 (10.0%)	9 (15.0%)	8 (13.3%)
Joint pain	4 (6.7%)	3 (5.0%)	2 (3.3%)	8 (13.3%)	5 (8.3%)	7 (11.7%)
Strength	5 (8.3%)	6 (10.0%)	3 (5.0%)	9 (15.0%)	5 (8.3%)	7 (11.7%)

The total HJHS scores had a wide span in all age groups and increased with age (*P* = 0.001). Except for the left elbow and the left knee joints, the proportion of abnormal HJHS scores in other four joints gradually increased with age (Table 4). Severity of disease, delayed treatment, type of treatment (on demand or prophylaxis), and high-intensity replacement therapy were not linked with an abnormal HJHS score (*P* > 0.05).

**Table 4:** The total HJHS score and the proportion distribution of each joint abnormality score in different age groups.

Age cohorts, year	4–8	8–14	14–18
Total HJHS score	0.00	5.00	9.00
Left elbow			
Normal	17 (85.0%)	19 (73.1%)	12 (85.7%)
Abnormal	3 (15.0%)	7 (26.9%)	2 (14.3%)
Right elbow			
Normal	17 (85.0%)	20 (76.9%)	10 (71.4%)
Abnormal	3 (15.0%)	6 (23.1%)	4 (28.6%)
Left knee			
Normal	16 (80.0%)	18 (69.2%)	10 (71.4%)
Abnormal	4 (20.0%)	8 (30.8%)	4 (28.6%)
Right knee			
Normal	17 (85.0%)	19 (73.1%)	6 (42.9%)
Abnormal	3 (15.0%)	7 (26.9%)	8 (57.1%)
Left ankle			
Normal	18 (90.0%)	19 (73.1%)	6 (42.9%)
Abnormal	2 (10.0%)	7 (26.9%)	8 (57.1%)
Right ankle			
Normal	17 (85.0%)	19 (73.1%)	5 (35.7%)
Abnormal	3 (15.0%)	7 (26.9%)	9 (64.3%)

#### 3.3.2 HEAD-US-C

**Figure 2:**
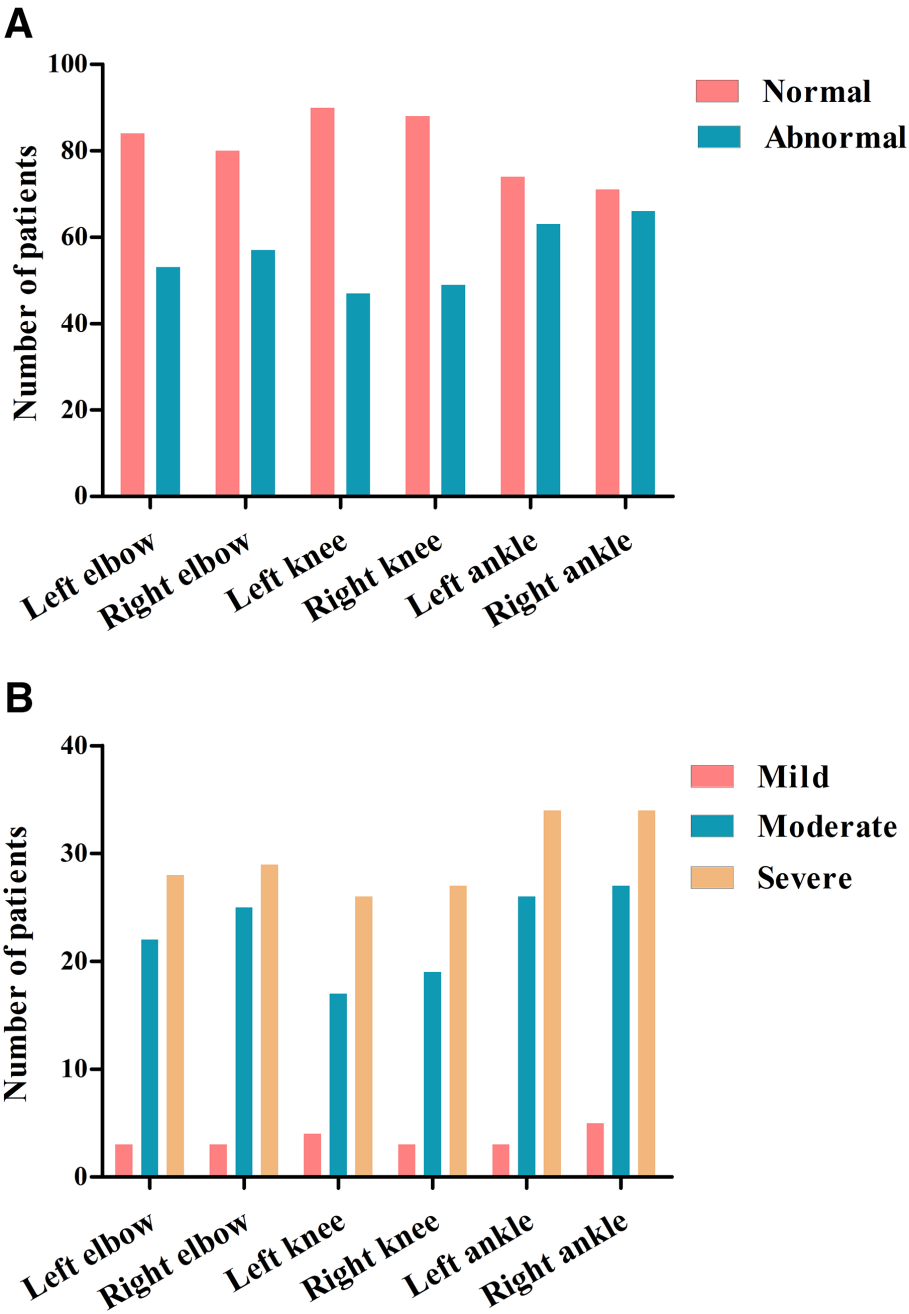
Joint assessments using HEAD-US-C in our cohort. (**A**) Numbers of abnormal joints identified by HEAD-US-C. (**B**) Numbers of abnormal joints in different severity of HB patients identified by HEAD-US-C.

In this study, 137 HB patients (822 joints), with a median age of 9.83 years, underwent HEAD-US-C assessment. Among them, 336 joints in 92 patients were affected, with the ankle joints being the most often affected (Figure 2). The median score of abnormal HEAD-US-C was 14.00 (ranging from 1 to 59). The age difference between the normal HEAD-US-C score group and the abnormal HEAD-US-C score group was statistically significant (*P* < 0.05). Synovial hyperplasia was more common in all joint lesions.

#### 3.3.3 Correlation analysis between the HJHS and HEAD-US-C

**Figure 3:**
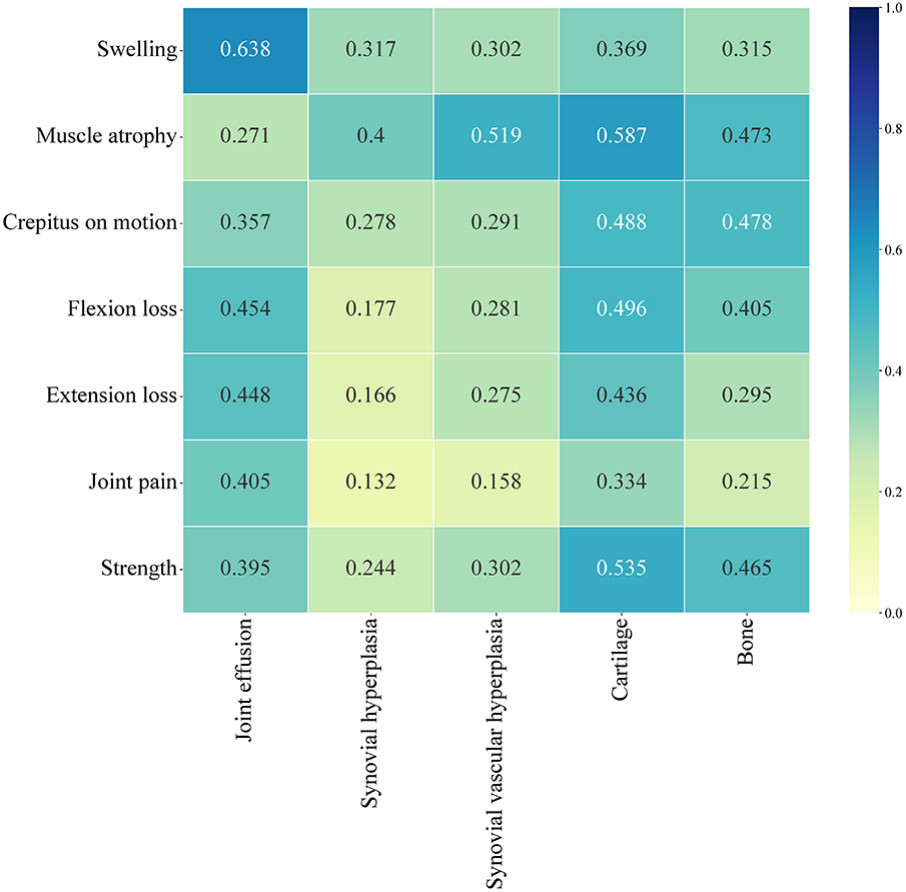
Correlation analysis between each dimension of HJHS and each dimension of HEAD-US-C.

A total of 56 patients completed both the HJHS and HEAD-US-C assessment. Among them, 23.2% (13/56) of the HB patients had HJHS score of 0 but positive HEAD-US-C score, while 7.1% (4/56) had positive HJHS score but HEAD-US-C score of 0. The HJHS score was significantly positively correlated with the HEAD-US-C (*r* = 0.542, *P* < 0.001). There was a strong correlation between swelling and joint effusion (*r* = 0.638). Muscle atrophy was moderately correlated with synovial hyperplasia (*r* = 0.400), synovial vascular hyperplasia (*r* = 0.519), cartilage loss (*r* = 0.587), and bone disease (*r* = 0.473). Flexion loss and extension loss were moderately associated with joint effusion (*r* = 0.454 and *r *= 0.448, respectively) and cartilage loss (*r* = 0.496 and *r *= 0.436, respectively). Crepitus on motion and muscle strength were moderately correlated with cartilage loss (*r* = 0.488 and *r* = 0.535, respectively) and bone disease (*r* = 0.478 and *r* = 0.465, respectively) (Figure 3).

### 3.4 QoL assessment

#### 3.4.1 SF-36

Fifty-two HB patients completed the SF-36 questionnaire, with a total mean (±SD) score of 52.12 (±18.85). The physical component summary (PCS) score was 47.07 (±18.96), and the mental component summary (MCS) score was 57.17 (±20.59). Among all eight dimensions evaluated, mental health yielded the best evaluation, followed by physical functioning and role-emotional functioning. In contrast, the general health dimension had the lowest average score. Table 5 presents the score of each dimension of SF-36.

**Table 5:** The total score and each dimension score of SF-36 in HB patients.

Parameters	Score (Mean ± SD)
Total	52.12 ± 18.85
Physical component summary	47.07 ± 18.96
Mental component summary	57.17 ± 20.59
Physical functioning	57.79 ± 27.36
Role-physical functioning	46.03 ± 27.23
Bodily pain	47.35 ± 20.93
General health	37.10 ± 22.41
Vitality	55.17 ± 21.39
Social functioning	53.61 ± 28.48
Role-emotional functioning	55.77 ± 29.03
Mental health	64.13 ± 19.62

Table 6 shows the relationship between the clinical characteristics of the HB patients with PCS, MCS and total SF-36 scores. Patients who received prevention treatment, without treatment delay and without high-intensity replacement therapy showed a significantly higher scores in the PCS (*P* = 0.023, *P* = 0.011, and *P* = 0.043, respectively) and total SF-36 (*P* = 0.021, *P* = 0.015, and *P* = 0.042, respectively). Patients who received prevention treatment and without treatment delay showed a significantly higher scores in the MCS (*P* = 0.033 and *P* = 0.036, respectively). Patients with inhibitors had lower total SF-36 score and PCS score than those without inhibitor (*P* = 0.039 and *P* = 0.021, respectively).

**Table 6:** Comparison of the total SF-36 score, total PSC score, and total MCS score in different groups of HB patients.

	No. of patients	Total SF-36			Physical component summary			Mental component summary		
		Score	*F*	*P*	Score	*F*	*P*	Score	*F*	*P*
Age group (year)			3.095	0.054		2.585	0.086		2.999	0.059
14–30	31	57.08 ± 18.04			51.75 ± 19.57			62.41 ± 18.70		
30–45	13	46.84 ± 15.52			41.53 ± 16.03			52.16 ± 15.88		
>45	8	41.47 ± 22.14			37.91 ± 16.98			45.03 ± 28.65		
Family history			0.393	0.533		0.024	0.876		1.022	0.317
Yes	25	50.40 ± 16.47			46.64 ± 16.25			54.17 ± 18.52		
No	27	53.71 ± 21.00			47.47 ± 21.48			59.95 ± 22.32		
Severity			0.352	0.556		0.535	0.468		0.171	0.681
Non-severe	24	50.67 ± 16.26			45.28 ± 16.55			56.07 ± 17.20		
Severe	24	53.81 ± 21.73			49.16 ± 21.62			58.46 ± 24.27		
First bleeding age (year)			1.277	0.293		1.107	0.365		1.194	0.326
≤1	19	54.37 ± 19.53			49.27 ± 21.43			59.48 ± 20.71		
1–3	15	46.99 ± 18.33			41.92 ± 17.42			52.06 ± 20.49		
3–8	7	64.09 ± 21.56			58.30 ± 20.24			69.87 ± 24.32		
8–14	4	44.55 ± 17.99			40.77 ± 17.73			48.34 ± 19.12		
>14	7	49.35 ± 13.01			44.48 ± 11.85			54.23 ± 15.14		
First bleeding inducement			1.090	0.302		0.646	0.425		1.373	0.247
Spontaneous bleeding	30	49.78 ± 18.28			45.25 ± 18.99			54.32 ± 20.02		
Non-spontaneous bleeding	22	55.30 ± 19.57			49.54 ± 19.09			61.06 ± 21.17		
First bleeding site			0.010	0.919		0.172	0.680		0.038	0.847
Mucocutaneous hemorrhage	25	52.40 ± 21.37			48.21 ± 20.91			56.59 ± 23.83		
Non-mucocutaneous hemorrhage	27	51.86 ± 16.59			46.01 ± 17.30			57.71 ± 17.50		
Diagnosis age (year)			0.880	0.483		1.227	0.312		0.611	0.657
≤1	16	57.60 ± 18.34			53.29 ± 18.04			61.92 ± 21.07		
1–3	2	48.27 ± 28.84			36.47 ± 21.52			60.06 ± 36.17		
3–8	13	52.11 ± 23.66			48.44 ± 22.54			55.78 ± 26.36		
8–14	6	55.62 ± 12.54			49.59 ± 15.13			61.65 ± 10.87		
>14	15	45.39 ± 19.07			39.64 ± 16.96			51.14 ± 16.13		
First treatment age			1.595	0.191		1.432	0.238		1.525	0.210
≤1	14	53.70 ± 20.29			47.95 ± 22.17			59.46 ± 21.29		
1–3	7	47.31 ± 27.22			41.41 ± 24.53			53.20 ± 30.96		
3–8	8	62.98 ± 16.50			56.46 ± 16.66			69.51 ± 17.97		
8–14	6	58.63 ± 14.35			55.81 ± 14.18			61.44 ± 15.15		
>14	17	45.39 ± 14.21			41.16 ± 14.58			49.61 ± 15.97		
Treatment delay			6.369	0.015*		7.035	0.011*		4.665	0.036*
Yes	28	46.31 ± 17.65			40.96 ± 18.20			51.66 ± 18.98		
No	24	58.90 ± 18.26			54.19 ± 17.62			63.60 ± 20.89		
Bleeding frequency (per year)			0.048	0.826		0.001	0.978		0.185	0.669
≤6	15	51.20 ± 14.70			47.18 ± 15.51			55.22 ± 15.92		
>6	37	52.49 ± 20.47			47.02 ± 20.39			57.96 ± 20.52		
Replacement therapy			5.704	0.021*		5.517	0.023*		4.791	0.033*
Prevention treatment	19	59.99 ± 19.09			54.87 ± 18.23			65.12 ± 21.78		
On-demand treatment	33	47.59 ± 17.42			42.58 ± 18.15			52.60 ± 18.69		
High intensity replacement therapy			4.345	0.042*		4.306	0.043*		3.580	0.064
Yes	30	47.60 ± 19.76			42.54 ± 19.67			52.66 ± 22.19		
No	22	58.28 ± 15.97			53.24 ± 16.42			63.33 ± 16.75		
Target joint			1.327	0.255		3.405	0.071		0.192	0.663
Yes	38	50.30 ± 19.67			44.19 ± 19.42			56.41 ± 21.98		
No	14	57.07 ± 16.03			54.88 ± 15.73			59.25 ± 16.75		
Inhibitor			4.516	0.039*		5.648	0.021*		2.889	0.095
Yes	6	37.24 ± 23.83			30.50 ± 26.43			43.98 ± 22.58		
No	46	54.06 ± 17.50			49.23 ± 16.99			58.89 ± 19.93		
Exposure day			0.890	0.417		0.715	0.494		0.926	0.403
<50	13	50.87 ± 13.42			46.75 ± 13.44			54.99 ± 14.55		
50–150	6	61.80 ± 9.41			55.72 ± 10.53			67.87 ± 12.36		
>150	33	50.85 ± 21.57			45.62 ± 21.72			56.08 ± 20.52		

* *P *< 0.05.

#### 3.4.2 CHO-KLAT 2.0

155 questionnaires, including 62 child-reported CHO-KLAT 2.0 and 93 parent-reported CHO-KLAT 2.0 were retrieved. Among them, 55 child-reported CHO-KLAT 2.0 had its corresponding parent questionnaires, and we observed a positive correlation between the child-reported CHO-KLAT 2.0 score and the parent-reported CHO-KLAT 2.0 score (*r *= 0.537, *P* < 0.001). The mean child-reported CHO-KLAT 2.0 score was 63.48 (±11.75) and 59.84 (±12.72) for parent-reported CHO-KLAT 2.0. It was found that CHO-KLAT 2.0 score was negatively correlated with age, and there was statistically significant difference in parent-reported CHO-KLAT 2.0 score of patients in different age groups (*r* = −0.256, *P* = 0.013). HB patients were grouped according to severity of disease, delayed diagnosis, delayed treatment, ABR, type of treatment, high-intensity replacement therapy, target joint, and inhibitor. There were no significant differences in either child-reported CHO-KLAT 2.0 score or parent-reported CHO-KLAT 2.0 score among these groups (*P *> 0.05).

The degree of correlation between the child-reported CHO-KLAT 2.0 and the parent-reported CHO-KLAT 2.0 with the overall evaluation of how much they were bothered by their hemophilia was −0.538 and −0.626, respectively (*P* < 0.001). Among them, children's self-perceived distress mainly included bleeding, exercise limitation, missing study due to bleeding, and injection. In addition, economic pressure, asymptomatic bleeding, and home care were also trouble their parents.

### 3.5 Genotype analysis

**Figure 4:**
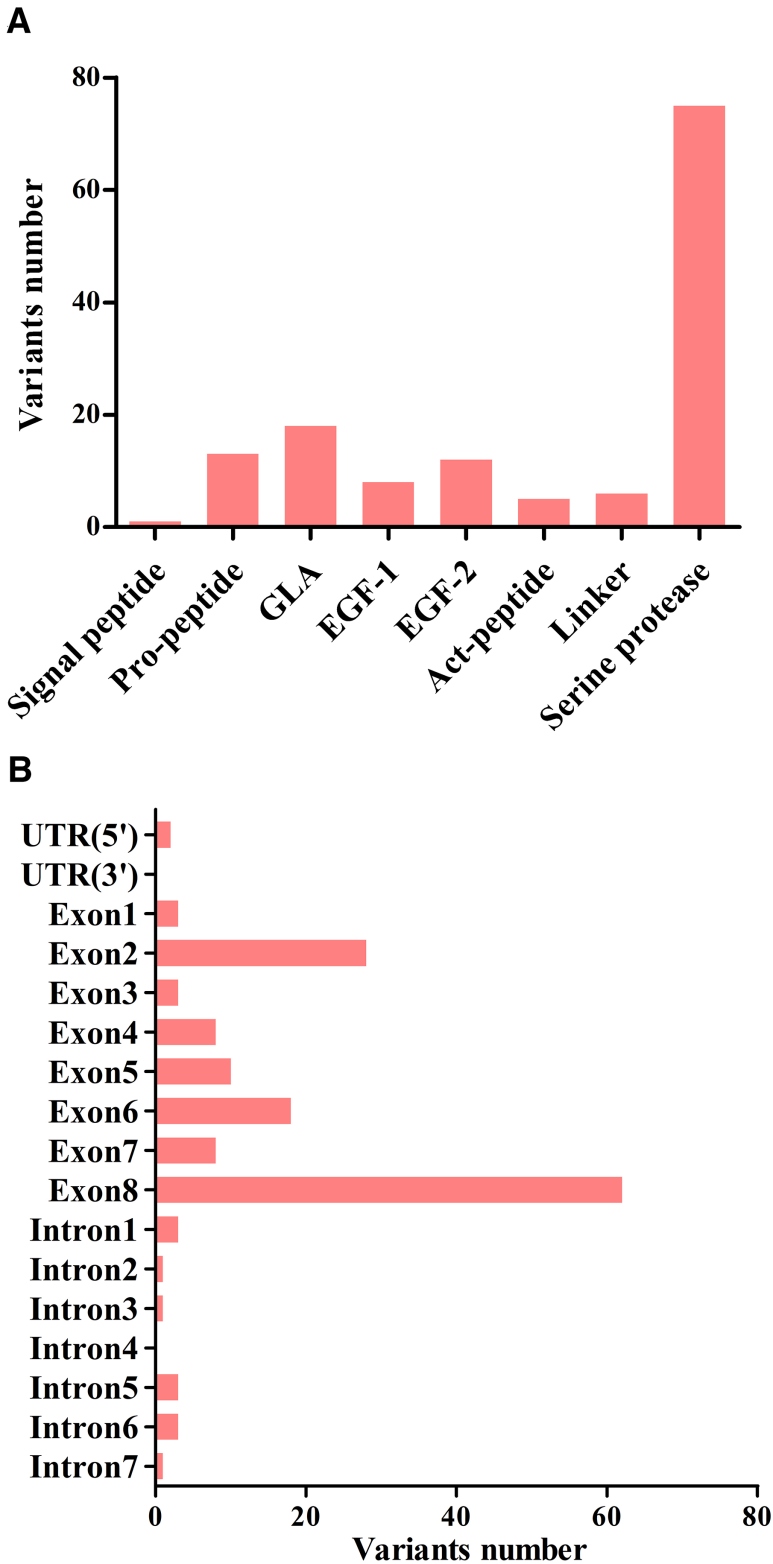
The distribution of point, duplication, and small deletion/insertion mutations (total 154 variants) throughout the FIX protein (**A**) and the *F9* gene (**B**).

A total of 123 unique mutations in the *F9* gene were found among the 158/185 HB patients, with point mutations being the most common (84.2%, 133/158), followed by deletion mutations (10.1%, 16/158) and deletion-insertion mutations (3.2%, 5/158). The majority of point mutations resulted in amino acid substitutions, which are known as missense mutations (63.2%, 84/133). 24.8% (33/133) of point mutations were nonsense mutations and caused premature termination of translation. Point mutations at CpG sites accounted for 30.1% (40/133), most of which were associated with Arg residues (95.0%, 38/40). Mutations in the serine protease domain accounted for 48.7% (75/154) of all domain mutations, and the majority of them were located in exon 8 (40.3%, 62/154) (Figure 4). In our study, eighteen mutations occurred in two or more patients. Combined with the *F9* variant database, most of them showed different severity (Table 7).

**Table 7:** Distribution of phenotypes in HB patients with the same mutation.

Variants	Severity, *n*				F9 variant database			
	Total	Mild	Moderate	Severe	Mild	Moderate	Severe	Unknown
c.1088G>A	2	0	0	2	0	1	1	2
c.1113C>G	2	0	0	2	0	1	2	1
c.1135C>T	5	0	2	3	2	7	52	14
c.1136G>A	5	0	4	1	4	54	15	7
c.127C>T	3	0	3	0	1	28	26	17
c.128G>A	7	0	3	4	3	25	59	11
c.1282delA	2	0	0	2	–	–	–	–
c.1292G>A	2	0	0	2	–	–	–	–
c.1306G>A	2	0	2	0	1	2	6	1
c.1358G>A	2	0	1	1	0	0	1	1
c.206G>A	2	0	0	2	0	0	7	2
c.223C>T	2	0	0	2	0	6	80	10
c.344A>C	2	1	1	0	–	–	–	–
c.572G>A	2	1	1	0	39	56	3	14
c.724-2A>G	2	0	0	2	0	1	1	0
c.874C>A	3	0	2	1	0	0	2	0
c.881G>A	3	0	3	0	16	92	16	16
c.892C>T	4	0	1	3	0	11	44	10

This study included 106 mothers of HB patients, and 84.0% (89/106) of these women were genetically identified as carriers. Coagulation factor activity was measured in 47 HB carriers. 19.1% (9/47) of them had FIX level ranging from 0.40 to 0.50 IU/ml, and 27.7% (13/47) of mild HB with FIX levels ranging from 0.05 to 0.40 IU/ml.

## 4 Discussion

A total of 185 HB patients (15 mild, 75 moderate, and 95 severe) were included in this study. The reason for low proportion of mild HB patients may be that mild HB patients generally do not have obvious bleeding when there is no trauma or surgery. However, for moderate and severe patients, the bleeding symptoms are often serious, which can be manifested as persistent bleeding after minor injury or spontaneous bleeding, so the treatment rate and diagnosis rate are high. 34.6% (64/185) and 38.5% (69/179) of HB patients had diagnostic delay and treatment delay. The reasons may be as follows: as a rare disease, the incidence of HB is low, and there is a lack of awareness among primary doctors. Primary hospitals did not have the required laboratory equipment for hemophilia diagnosis, resulting in diagnostic delay. Hemophilia knowledge was rarely preached, and parents' understanding of the disease was weak. Besides, some parents did not pay enough attention to the slight bleeding symptoms of patients, which led to the delay of diagnosis and treatment.

In terms of the first treatment, the most popular first-line drug used was PCC, accounting for 50.8% (91/179) of patients. This suggests that PCC is widely recognized and utilized as an effective treatment for managing the first bleeding episodes. The content of FⅨ is the international standard for the efficacy of PCC, with 1 IU of FⅨ is equivalent to the activity of FⅨ in 1 ml of fresh frozen plasma (FFP), and the concentration of total coagulation factors in the PCC is equivalent to 25 times that in concentrated human plasma (25). Cryoprecipitate, a FⅧ-rich product, is used for patients with hemophilia A. Because of the high incidence of hemophilia A, four patients were first treated with cryoprecipitate when only prolonged APTT was known. Bleeding phenotype in PWH is generally related to the residual factor levels. Recurrent joint bleeding can cause long-term complications including pain, arthropathy, and disability. Therefore, the main goal of treatment of PWH is to reduce the frequency of bleeding and thus mortality and joint damage. Timely detection of bleeding and replacement of missing coagulation factors (on demand) or prevention through routine prophylaxis is the basis of treatment (26). However, the immune system response to foreign antigens introduced during replacement therapy can produce inhibitors that lead to neutralization of coagulation factor activity. In our study, the incidence of inhibitors was 6.1% (11/179), which is in line with previous reports (27)^.^

In this study, 71.4% (132/185) of HB patients had a history of joint bleeding, and the incidence of target joint was 45.9% (85/185). Hemophilic arthropathy is the most serious consequence of hemophilia and the leading cause of decreased QoL. Currently, the biggest challenges are uncertainties about how to rehabilitate existing joint damage in individuals with musculoskeletal problems and difficulties in accessing expert physiotherapists in hemophilia (28). Thus, to minimize joint damage and enhance the QoL for hemophilia patients, it is essential to assess joint function, identify joint bleeding, and implement appropriate treatment strategies as soon as feasible.

HJHS provides clinicians a specific, reliable and effective method to monitor joint status at any time (21). In our study, 60 HB patients underwent HJHS assessment, and 58.3% (35/60) of them had abnormal HJHS scores. With the increase of age, the proportion of abnormal HJHS score increased, which may be related to the increase of weight, physical activity, and damage due to previous joint bleeds. In recent years, musculoskeletal ultrasound has become a primary diagnostic tool for joint damage in patients with hemophilia because of its simplicity, low price, no radiation and sensitivity to soft tissue and cartilage lesions (29). The HEAD-US-C scale was utilized to assess joint structure in 137 HB patients in this study. The most common site of joint lesions was ankle joint, followed by elbow joints and knee joints, which was consistent with the studies based on Pettersson score in Taiwan (30). It was considered to be due to the ankle joint bleeding caused by running, jumping and other sports in childhood, and repeated bleeding can cause irreversible damage to the ankle joint. The HJHS score was positively correlated with the HEAD-US-C score, that is, the more serious the joint structure lesions detected by ultrasound, the worse the joint function, which was consistent with previous studies. However, objective measurement of joint health through physical examination remains a major challenge to the medical team.

The SF-36 and CHO-KLAT are two commonly used scale to assess the QoL of patients. In our study, the total mean (±SD) score of SF-36 was 52.12 (±18.85), which was higher than that reported by Hosseini (44.72 ± 20.20) and Haghpanah (49.26 ± 21.57), with the general health domain had the lowest average scores. This may be due to differences in social determinants of health and culture among different countries (31, 32). The mean child-reported CHO-KLAT 2.0 score and parent-reported CHO-KLAT 2.0 score were 63.48 (±11.75) and 59.84 (±12.72), respectively, which were lower than 74.6 ± 14.0 and 74.5 ± 11.6 reported by Bradley (33). The reason may be that the standard treatment of children abroad, including early diagnosis, prophylaxis treatment, can effectively improve the QoL of patients. In addition, with the increasing of age, the CHO-KLAT 2.0 (parents' book) score decreased. It's possible that with the increase of age, weight and the expansion of daily activities, the patients were out of their parents' vision, and the dosage and frequency of medication were also increased, and the economic and psychological burden reduced the parents' happiness. As a result, the QoL of the parents is reduced.

The identification of gene mutation is helpful in identifying the severity of disease and predicting the risk of inhibitor formation. In this study, a total of 123 variations were identified among the 158 HB patients, which might be explained by recurrent mutations (RM) or mutations shared by individuals with the identical by descent (founder effect) (34). The CpG islands, known for their high levels of methylation on cytosine residues, have been identified as hotspots for the “C→T” transitions (35). Arginine was found to be particularly susceptible to mutation as four of its codons contain CpG dinucleotides (36). In terms of the frequency of mutation types, our findings align with the data in the *F9* variant database (3). Further investigations have revealed that mutations in the serine protease domain were more frequently observed (37). Amongst these mutations, the most common alteration, accounting for 40.3% (62/154) of the cases, occurred in exon 8. This particular exon is believed to be related to the catalytic domain of the *F9* protein, further emphasizing its importance in the functioning of the gene.

Several studies have shown two special heterogeneities in hemophilia patients. Patients with the same levels of FⅨ: C may have different clinical manifestations, that is, phenotypic heterogeneity. The other is genotype-phenotype heterogeneity, where the same genotype exhibits different severity of disease (37, 38). As shown in Table 7, the missense mutation, c.1136G>A (p. Arg379Gln), was observed in five patients, of whom 4 patients were moderate and 1 patient was severe. This mutation has been reported 80 times in the *F9* variants database and is association with mild, moderate, and severe phenotype.

Our study analyzed the demographics, clinical manifestations, diagnostic and treatment characteristics, inhibitor incidence, genotype, joint status and QoL of patients with HB in the central and western regions of China, as well as *F9* gene and factor activity levels of their mothers, to provide a basis for hemophilia management.

## Data Availability

The original contributions presented in the study are included in the article/Supplementary Materials, further inquiries can be directed to the corresponding author.

## Appendix

**Appendix 1:** Hemophilia joint health score version 2.1 (HJHS 2.1).

Swelling
0 = none
1 = mild
2 = moderate
3 = severe
Duration of swelling
0 = no swelling or <6 months
1 = > 6 months
Muscle atrophy
0 = none
1 = mild
2 = severe
Crepitus on motion
0 = none
1 = mild
2 = severe
Flexion loss
Comparison with the contralateral side
0 = < 5°
1 = 5–10°
2 = 11–20°
3 = > 20°
Comparison with normal values
0 = within the normal range
1 = 1–4°
2 = 5–10°
3 = > 10°
Extension loss
Comparison with the contralateral side
0 = < 5°
1 = 5–10°
2 = 11–20°
3 = > 20°
Comparison with normal values
0 = within the normal range
1 = 1–4°
2 = 5–10°
3 = > 10°
Joint pain
0 = no pain either through the active range of motion
1 = no pain throughout the active range of motion, except for mild overpressure or palpation
2 = Pain throughout the active range of motion
Strength
0 = holds test position against gravity with maximum resistance (gr. 5)
1 = holds test position against gravity with moderate resistance (but breaks with maximal resistance) (gr. 4)
2 = holds test position with minimal resistance (gr. 3+), or holds test position against gravity(gr. 3)
3 = able to partially complete ROM against gravity (gr. 3−/2+), or able to move through ROM gravity eliminated (gr. 2), or through partial ROM gravity eliminated
4 = trace (gr. 1) or no muscle contraction (gr.0)
NE = unable to assess
Global gait score (walking, stairs, running, skipping)
0 = all skills are within normal limits
1 = one skill is not within normal limits
2 = two skills are not within normal limits
3 = three skills are not within normal limits
4 = no skills are within normal limits

Joint: including left elbow, right elbow, left knee, right knee, left ankle, and right ankle.

Total score = Joint totals + Global gait score.

**Appendix 2:** Hemophilia early arthropathy detection with ultrasound in China (HEAD-US-C).

Joint effusion
0 = none
1 = small (3–9 mm)
2 = medium (10–19 mm)
3 = large (≥20 mm)
Synovial hyperplasia
0 = none
1 = mild (no more than the line of the highest point of the bone surface)/moderate (no more than the line of the highest point of the bone surface, but no more than the diaphasis)
2 = severe (exceeding the line of the highest point of the bone surface and extending beyond the diaphysis on one side)
Synovial vascular hyperplasia
0 = none
1 = ROI blood flow signal less than 3
2 = ROI blood flow signal greater than or equal to 3 places/dendritic blood flow signal
Cartilage
0 = normal
1 = loss of less than 25% of articular cartilage on the target surface
2 = loss of less than or equal to 50% of articular cartilage on the target surface
3 = loss of more than 50% of articular cartilage on the target surface
4 = complete absence of articular cartilage on the target surface
Bone
0 = normal
1 = mild subchondral bone irregularity with/without periarticular small osteophytes
2 = significant irregularity of subchondral bone and/or significant periarticular osteophyte formation

Joint: including left elbow, right elbow, left knee, right knee, left ankle, and right ankle.
